# Effectiveness of a Post-Acute-Care Rehabilitation Program in Patients with Stroke: A Retrospective Cohort Study

**DOI:** 10.3390/life15081216

**Published:** 2025-08-01

**Authors:** Yi-Pang Lo, Mei-Chen Wang, Yao-Hsiang Chen, Shang-Lin Chiang, Chia-Huei Lin

**Affiliations:** 1 Department of Nursing, Tri-Service General Hospital Songshan Branch, Taipei 10581, Taiwan; winfly1017@gmail.com (Y.-P.L.); angel0937303771@yahoo.com.tw (M.-C.W.); 810792me@gmail.com (Y.-H.C.); 2 Graduate Institute of Medical Sciences, College of Medicine, National Defense Medical University, Taipei 11490, Taiwan; 3 Department of Physical Medicine and Rehabilitation, Tri-Service General Hospital, Taipei 11490, Taiwan; andyyy520@yahoo.com.tw; 4 College of Medicine, National Defense Medical University, Taipei 11490, Taiwan; 5 College of Nursing, National Defense Medical University, Taipei 11490, Taiwan

**Keywords:** post-acute care, subacute care, stroke, rehabilitation, muscle strength, physical functional performance

## Abstract

Early rehabilitation is essential for restoring functional recovery in patients with stroke, particularly during the early phase of post-acute care (PAC), or the subacute stage. We aimed to evaluate the effectiveness of a 7-week PAC rehabilitation program in improving muscle strength, physical performance, and functional recovery. A total of 219 inpatients with stroke in the subacute stage were initially recruited from the PAC ward of a regional teaching hospital in Northern Taiwan, with 79 eligible patients—within 1 month of an acute stroke—included in the analysis. The program was delivered 5 days per week, with 3–4 sessions daily (20–30 min each, up to 120 min daily), comprising physical, occupational, and speech–language therapies. Sociodemographic data, muscle strength, physical performance (Berg Balance Scale [BBS], gait speed, and 6-minute walk test [6MWT]), and functional recovery (modified Rankin Scale [mRS], Barthel Index [BI], Instrumental Activities of Daily Living [IADL], and Fugl–Meyer assessment: sensory and upper extremity) were collected at baseline, 3 weeks, and 7 weeks. Generalized estimating equations analyzed program effectiveness. Among the 56 patients (70.9%) who completed the program, significant improvements were observed in the muscle strength of both the affected upper (B = 0.93, *p* < 0.001) and lower limbs (B = 0.88, *p* < 0.001), as well as in their corresponding unaffected limbs; in physical performance, including balance (BBS score: B = 9.70, *p* = 0.003) and gait speed (B = 0.23, *p* = 0.024); and in functional recovery, including BI (B = 19.5, *p* < 0.001), IADL (B = 1.48, *p* < 0.001), and mRS (B = −0.13, *p* = 0.028). These findings highlight the 7-week PAC rehabilitation program as an effective strategy during the critical recovery phase for patients with stroke.

## 1. Introduction

Stroke is the second leading cause of death and the third leading cause of disability-adjusted life years (DALYs) worldwide. In 2021, an estimated 12.7 million people experienced a new stroke and 6.6 million died from stroke-related causes. Stroke often results in long-term disability, particularly affecting motor function and daily activities [1]. Globally, approximately 15 million people experience a stroke each year, with around 5 million left permanently disabled [2].

In addition to physical impairments, stroke affects mental health. Physical sequelae may include limb weakness, sensory deficits, aphasia, dysphagia, hemineglect, coordination problems, and cognitive impairment [3]. These impairments are often accompanied by various psychological challenges [4]. According to a recent systematic review and meta-analysis, depression is the most frequent psychiatric condition after stroke and is closely associated with adverse health outcomes. Based on pooled data from 77 studies, the overall prevalence of poststroke depression was estimated to be 27%, with many cases emerging within the first three months after stroke onset [5]. Moreover, suicide accounts for approximately 3–4 deaths per 1000 stroke survivors within the first 5 years of the event [6]. These findings highlight the need for an integrated interdisciplinary approach to rehabilitation programs, emphasizing early intervention to provide comprehensive care and improve outcomes for individuals recovering from stroke [4].

Early rehabilitation plays a critical role in promoting functional recovery after stroke, especially during the early post-acute phase, which corresponds to the subacute stage and is often regarded as the “golden period” of recovery. A recent meta-analysis of 16 randomized controlled trials, involving 1908 patients with ischemic stroke, demonstrated that early rehabilitation compared to delayed intervention resulted in significantly greater improvements in functional independence, motor recovery, and neurological function, as reflected in higher Barthel Index, Fugl–Meyer assessment scale, and reduced NIHSS scores [7]. A recent randomized controlled trial found that early rehabilitation combined with virtual reality training in patients with first-time acute stroke led to greater improvement in psychological health, particularly in reducing depressive symptoms [8]. These findings suggest that while early rehabilitation is beneficial, its effectiveness may vary depending on the specific components included in the intervention.

Many patients continue to experience functional impairments after acute medical treatment and often cannot return directly to their homes or communities, underscoring the need for a transitional care model to bridge the gap between hospital discharge and community reintegration. The post-acute-care (PAC) model for stroke includes various options, such as inpatient rehabilitation facilities, skilled nursing facilities, and home health agencies that provide essential services [9]. This model plays a vital role in supporting a smooth transition by addressing both physical and mental health needs during the recovery phase. In Taiwan, the PAC rehabilitation program for stroke survivors provides a structured 6–8-week regimen that includes physical, occupational, and speech–language therapies. This comprehensive approach facilitates the transition from acute inpatient care to community living and aims to optimize functional and psychosocial outcomes [4]. Although the benefits of early rehabilitation are well established, the short-term effectiveness of a structured PAC rehabilitation program remains unexplored. Most existing studies focus on long-term outcomes, with limited attention to targeted interventions during the critical early recovery period. There is limited evidence assessing the comprehensive, multidisciplinary approach applied in real-world PAC programs, particularly during the subacute phase of stroke recovery. Moreover, few studies have examined short-term functional outcomes, despite the clinical importance of this transitional period. This creates a gap in the literature regarding the practical effectiveness of integrated PAC models for stroke. Additionally, although a PAC rehabilitation program for patients with stroke has been implemented in Taiwan since 2014, evidence evaluating its impact on muscle strength, physical performance, and functional recovery remains limited.

Therefore, this study aimed to evaluate the short-term effects of a structured PAC rehabilitation program for patients with stroke. We hypothesized that participation in the PAC program would significantly improve muscle strength, physical performance, and functional recovery. The primary research question was as follows: does a 7-week PAC rehabilitation program lead to measurable improvements in these outcomes during the post-acute phase of stroke recovery?

## 2. Materials and Methods

### 2.1. Research Design

This retrospective cohort study was conducted in a PAC ward of a regional teaching hospital in Northern Taiwan. The study followed the Strengthening the Reporting of Observational Studies in Epidemiology guidelines.

### 2.2. Participants

A total of 219 eligible participants were screened through chart review and confirmed by a research nurse and a physician. The inclusion criteria were as follows: (1) Occurrence of an acute stroke event within the past month. (2) A medically stable condition, defined as no neurological deterioration for at least 72 h, stable or manageable vital signs (including blood pressure, heart rate, and temperature) for at least 72 h, and resolution or effective management of complications, such as infection, hematological abnormalities, or gastrointestinal bleeding. (3) A functional status corresponding to a modified Rankin scale (mRS) score of 3–4. (4) Potential for active rehabilitation as determined by the medical team, based on adequate cognitive function, learning ability, and motivation; sufficient physical endurance to tolerate at least 1 h in a seated position (with or without support) in a wheelchair or at the bedside; and a willingness to actively participate in the rehabilitation program.

The exclusion criteria were as follows: (1) ongoing intravenous antibiotic therapy due to acute infection or continued contact isolation precautions for multi-drug-resistant organism infection beyond 30 days post-stroke onset; (2) use of anticoagulants beyond 30 days post-stroke onset without achieving or maintaining an appropriate therapeutic international normalized ratio (target range: 1.5–3.0); (3) persistent poor glycemic control—defined as fasting blood glucose levels >200 mg/dL—requiring close monitoring despite appropriate treatment with insulin or oral hypoglycemic drugs for more than 4 weeks after stroke onset; (4) prolonged intensive care unit stay exceeding 30 days; and (5) prolonged hospitalization due to serious complications or comorbidities (e.g., myocardial infarction, venous thromboembolism, or acute kidney injury) requiring specialized interventions or surgical treatment beyond 30 days after stroke onset.

A priori power analysis was performed using G*Power 3.1 to estimate the required sample size for detecting a moderate effect size (dz = 0.5) with a paired-sample *t*-test. Assuming a two-tailed test, α = 0.05, and statistical power = 0.80, the analysis indicated a minimum sample size of 34 participants would be required [10].

Participants were categorized into two groups based on the actual duration of their participation in the PAC program. Those who completed the entire 7-week program as initially prescribed were assigned to the completed group. In contrast, participants who discontinued the program before 7 weeks—due to early achievement of rehabilitation goals, medical complications, transfer to other facilities, or personal decisions—were classified as the discontinued group. This grouping was determined retrospectively using electronic medical records and discharge notes.

### 2.3. PAC Rehabilitation Program

In 2014, Taiwan’s National Health Insurance Administration launched the PAC for Cerebrovascular Disease program, which supports the transfer of patients with medically stable stroke to designated rehabilitation hospitals within 30 days of stroke onset, provided that they are willing to participate in intensive rehabilitation [11]. The 7-week PAC rehabilitation program adopts a comprehensive, interdisciplinary approach involving physicians, neurologists, physical and occupational therapists, speech–language pathologists, nutritionists, and nurses. This team convenes daily to monitor patient progress and adjust treatment plans accordingly [12]. During the initial 24–48 h following admission, patients received intensive therapeutic sessions approximately every hour, establishing a strong foundation for recovery.

The program focused on promoting functional recovery, enhancing motor skills, fostering independence in daily activities, and improving physical performance through balance, gait, strength, and endurance training. In the inpatient setting, intensive rehabilitation was provided for 3–4 sessions per day, 5 days per week, for 7 weeks. Each session lasted 20–30 min, with a total of up to 120 min per day. The individualized program was based on the patient’s functional goals and assessed needs, in accordance with the FITT-VP principles (Frequency, Intensity, Time, Type, Volume, and Progression). The progress of the program was continuously monitored and adjusted accordingly to ensure safety and optimize outcomes. Cognitive function was actively addressed through stimulation and targeted interventions for identified deficits. Nutritional needs and swallowing difficulties were managed through dietary counseling and strategies to improve swallowing function. The program also prioritized psychosocial well-being by offering emotional and social support to facilitate community reintegration. Specific training in occupational performance—including self-care and work-related skills—helped patients regain independence.

Physicians and neurologists played a central role in patient management, including overall medical supervision, diagnosis confirmation, rehabilitation goal setting, and ongoing evaluation of neurological recovery. They also participated in the daily interdisciplinary meetings to adjust individualized rehabilitation plans based on patient progress. Additional treatments provided during the program included spasticity management (e.g., oral medications or botulinum toxin injections when necessary), pain control, prevention of complications (e.g., deep vein thrombosis prophylaxis), and management of comorbid conditions. Orthoses, such as ankle-foot orthoses, wrist-hand orthoses, or slings, were prescribed as needed by rehabilitation specialists to support limb positioning, promote mobility, and facilitate safe ambulation.

### 2.4. Data Collection

#### 2.4.1. Sociodemographic and Lifestyle

Sociodemographic and lifestyle data were retrospectively collected from medical records. Variables included age, sex, marital status, religious beliefs, current employment status, surgical history, household composition, smoking habits, and alcohol consumption. Information about the primary caregiver and the length of hospital stay prior to enrollment in the PAC program was also recorded. Additionally, the presence of comorbidities, such as hypertension, heart disease, type 2 diabetes, or lipidemia, was documented.

#### 2.4.2. Outcome Indicators

The individualized rehabilitation program followed a standardized assessment process, with data retrospectively collected from patients’ medical records. Evaluations included muscle strength; physical performance (Berg Balance Scale [BBS], gait speed, and 6-minute walk test [6MWT]); functional recovery (mRS, Barthel Index [BI], Instrumental Activities of Daily Living [IADL] scale, and Fugl–Meyer assessment [FMA]); nutritional status (functional oral intake scale [FOIS] and mini nutritional assessment [MNA]); cognitive function (mini-mental state examination [MMSE]); occupational performance; and language ability (concise Chinese aphasia test [CCAT]). Assessments were conducted at three time points: baseline, 3 weeks, and 7 weeks after initiation of the PAC program. All physical assessments, including muscle strength testing, were conducted during the subacute stage of stroke—the early phase of PAC—after patients had achieved medical stability and were able to reliably follow instructions. No assessments were conducted during the hyperacute or unstable phase of stroke.

#### 2.4.3. Muscle Strength

Muscle strength was assessed using the Medical Research Council (MRC) Oxford Scale, a six-point ordinal scale ranging from 0 (no contraction) to 5 (normal strength). Despite being primarily a clinical tool, the Oxford Scale is widely used in stroke rehabilitation research due to its ease of administration, ability to assess key muscle groups, and strong inter-rater reliability (intraclass correlation coefficient, ICC = 0.85–0.96) [13,14]

#### 2.4.4. Physical Performance

(1) Berg Balance Scale (BBS)

The BBS (range: 0–56) assesses balance ability, with higher scores indicating greater stability. It has demonstrated good predictive validity for fall risk in stroke populations. A logistic regression model showed that BBS scores at admission were significantly associated with fall incidence, with a cut-off score ≤ 29 yielding a sensitivity of 80% and specificity of 78% for identifying fallers [15]. This supports its clinical utility and criterion validity as a balance assessment tool in post-stroke rehabilitation.

(2) Gait speed

Gait speed, a significant prognostic factor for mortality [16]. It is also a valid and reliable measure of post-stroke ambulation, supported by high test–retest reliability. Specifically, the ICC of gait speed has been reported as 0.94, indicating excellent measurement stability across repeated assessments [17].

(3) 6-Minute Walk Test (6MWT)

The 6MWT provides a simple and widely adopted assessment of functional capacity in individuals with stroke and demonstrates high test–retest reliability [18]. The 6MWT has demonstrated excellent test–retest reliability, with an ICC of 0.99, supporting its robustness and clinical applicability in stroke rehabilitation settings [18].

#### 2.4.5. Functional Recovery

(1) Modified Rankin Scale (mRS)

The mRS is a widely used 7-point scale used for assessing post-stroke disability, with scores ranging from 0 (no symptoms) to 6 (death). Scores of 0–3 indicate mild to moderate disability, whereas scores of 4–5 indicate severe disability. It has been validated for use in both clinical trials and routine practice. It has demonstrated moderate to strong inter-rater reliability, which significantly improves when structured interviews are used (κ = 0.56 without vs. κ = 0.78 with structured interviews). The test–retest reliability is also strong, with κ values ranging from 0.81 to 0.95, indicating high stability across repeated assessments [19,20].

(2) Barthel Index (BI)

The BI evaluates functional independence in 10 basic activities of daily living, including self-care and mobility, with total scores ranging from 0 to 100 in 5-point increments. Higher scores indicate greater independence. The BI has demonstrated good to excellent internal consistency (Cronbach’s α = 0.80–0.93), good test–retest reliability, and moderate to very good inter-rater reliability (Cohen’s κ = 0.41–1.00) in stroke populations. It also shows strong content, construct, concurrent, and predictive validity, supporting its widespread use in stroke rehabilitation research [21].

(3) Instrumental Activities of Daily Living (IADL)

The IADL scale, widely used for monitoring functional status in community and clinical settings, was used to evaluate higher-level daily functions, such as shopping, cooking, and managing finances. It includes 8 items scored from 0 (independent) to 1 (dependent), with a total score ranging from 0 to 8. The scale demonstrates high inter-rater reliability (ICC = 0.85), moderate test–retest reliability (ICC = 0.75), and good internal consistency (Cronbach’s α = 0.90), supporting its validity and utility in evaluating functional capacity among stroke survivors [22,23,24].

(4) Fugl–Meyer assessment (FMA)

The FMA is a validated, stroke-specific tool for assessing motor and sensory recovery. It employs a 3-point scale (0–2) and includes 155 items across 5 domains. In this study, only the upper extremity motor (maximum score = 66) and sensory (maximum score = 24) subscales were used. These were administered by a trained therapist in approximately 30 min. The FMA, a core assessment tool in Taiwan’s PAC programs, exhibits excellent psychometric properties, including high intra-rater (ICC = 0.98) and inter-rater reliability (ICC = 0.95), strong internal consistency (Cronbach’s α > 0.96), and well-established construct and criterion validity compared with other standardized motor assessments [25,26,27].

#### 2.4.6. Nutritional Status

(1) Functional Oral Intake Scale (FOIS)

The FOIS (score range: 1–7) is a reliable and valid tool used to assess functional oral intake in patients with stroke, with higher scores indicating better swallowing ability. The FOIS demonstrates high inter-rater reliability (Cohen’s κ = 0.86–0.91), as well as strong consensual validity (0.90) and high criterion validity at both stroke onset and one month post-stroke [28].

(2) Mini Nutritional Assessment (MNA)

The MNA (score range: 0–30) is a validated tool for evaluating nutritional status, with higher scores indicating better nutritional health. Scores ≥ 24 indicate normal nutritional status, 17–23.5 suggest risk of malnutrition, and <17 reflect protein-energy malnutrition. The MNA demonstrates high sensitivity (96%), specificity (98%), and positive predictive value (97%) compared with clinical evaluations. It also shows strong inter-rater reliability and good criterion and construct validity through correlations with serum albumin and relevant clinical outcomes [29].

#### 2.4.7. Cognitive Function

The mini-mental state examination (MMSE), a widely used cognitive screening tool (score range: 0–30), has demonstrated moderate-to-high reliability and validity, with sensitivity ranging from 71% to 92% and specificity from 56% to 96%. It also shows good construct validity through its correlations with functional and cognitive outcomes [30,31].

#### 2.4.8. Occupational Performance

Occupational performance was assessed using the Motor Activity Log (MAL), a semi-structured interview assessing the use of the affected upper limb in daily life. It includes two domains: amount of use (AOU) and quality of movement (QOM), each rated from 0 to 5. Higher scores indicate more frequent and higher-quality use of the paretic limb. The MAL demonstrated high internal consistency (AOU: Cronbach’s α = 0.88; QOM: α = 0.91), moderate construct validity (Spearman’s ρ = 0.63), good agreement limits, and strong responsiveness (ratios of 1.9 for AOU and 2.0 for QOM) [32].

#### 2.4.9. Language Ability

Language ability was assessed using the concise Chinese aphasia test (CCAT), a standardized tool for evaluating aphasia in Chinese-speaking individuals post-stroke. The CCAT assesses speaking, comprehension, reading, and writing abilities, with scores ranging from 1 to 12—higher scores indicating better language function. The test demonstrates excellent reliability, with split-half reliability coefficients above 0.90, test–retest reliability ranging from 0.93 to 0.99, and inter-rater reliability between 0.88 and 0.99 [33].

### 2.5. Ethical Consideration

Ethical approval for this study was granted by the Institutional Review Board of the local medical center (TSGHIRB: B202205027).

### 2.6. Data Analysis

Statistical analyses were performed using SPSS (version 24.0; IBM Corp., Armonk, NY, USA). Descriptive statistics, including means and standard deviations (SDs) or number (n) and percentages (%), were used to summarize participant characteristics. Group comparisons (completed vs. discontinued PAC program) for sociodemographic and baseline characteristics were conducted using chi-square tests (*x*^2^), Student’s *t*-tests (*t*), or Fisher’s exact tests, as appropriate. Between-group differences in outcome indicators at baseline and 3 weeks were evaluated using the Mann–Whitney U test (Z), due to potential deviations from normality in several outcome measures. The completed PAC program group was assessed at both 3 and 7 weeks post-baseline, while the withdrawn group was assessed only at 3 weeks. To assess the effectiveness of the PAC program, a generalized estimating equation (GEE) analysis for longitudinal data was performed, accounting for the interaction between group and time (group × time). The GEE approach, originally proposed by Liang and Zeger, extends generalized linear models to accommodate correlated or repeated observations, allows adjustment for multiple covariates, and manages outcome variables that are not normally distributed [34]. The model was adjusted for potential covariates including age, sex, marital status, caregiver, religious beliefs, current employment status, history of surgery, living arrangement, stroke type, smoking, alcohol consumption, and comorbidities (e.g., hypertension, heart disease, type 2 diabetes, and dyslipidemia). Statistical significance was set at *p* < 0.05.

## 3. Results

### 3.1. Participant Characteristics

**Figure 1:**
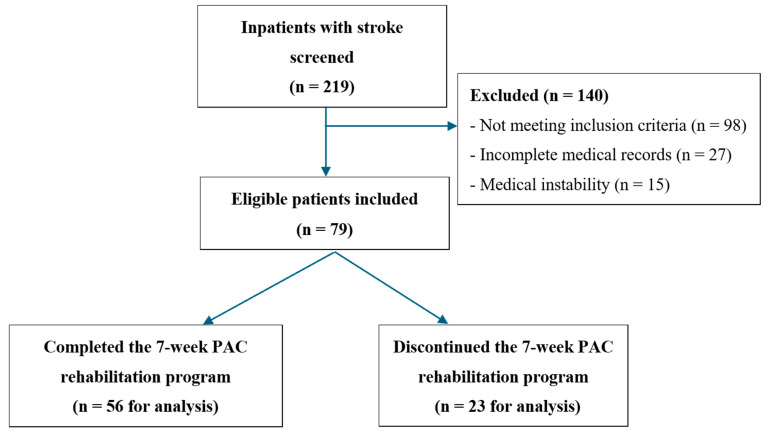
Flowchart of the patient enrollment process in the study.

A total of 219 inpatients with stroke were initially screened for eligibility through a review of medical records. Among these, 140 patients were excluded for the following reasons: not meeting the inclusion criteria (*n* = 98), incomplete medical records (*n* = 27), or presenting with medical instability (*n* = 15). Specifically, patients were excluded if they (1) required ongoing intravenous antibiotic treatment or contact isolation due to infection beyond 30 days post-stroke onset, (2) had unstable anticoagulation status without achieving therapeutic INR, (3) exhibited persistent poor glycemic control despite medication, (4) experienced prolonged ICU stays exceeding 30 days, or (5) had extended hospitalization due to serious comorbidities requiring specialized care. After applying these criteria, 79 eligible patients were included in the study, as illustrated in Figure 1.

A total of 56 patients (70.9%) completed the 7-week PAC program, while 23 (29.1%) discontinued the PAC program for various reasons, including death (*n* = 2), clinical deterioration requiring intensive care unit admission (*n* = 3), and sufficient functional improvement allowing discharge to home (*n* = 18). The majority of participants had ischemic stroke (82.3%), were men (53.2%), married (79.7%), and older adults (mean age: 67.35 years). Most patients lived with their families (89.9%), and the mean length of hospital stay prior to PAC program enrollment was 35 days (SD = 9.93). Primary caregivers included family members (48.1%), foreign domestic workers (26.6%), and professional caregivers (25.3%) (Table 1).

### 3.2. Comparisons of Sociodemographic and Disease Characteristics Between Patients Who Completed and Those Who Discontinued the Pac Rehabilitation Program

To explore potential factors associated with adherence to the PAC program, we compared baseline sociodemographic and disease characteristics between patients who completed the program and those who discontinued. Identifying these differences may help clinicians better understand which patients are more likely to complete structured rehabilitation and tailor interventions accordingly.

Table 1 presents a comparison of sociodemographic and baseline characteristics between the 56 patients who completed and 23 patients who discontinued the PAC rehabilitation program. No significant differences were observed between the two groups in terms of age, sex, caregiver type, marital status, religious beliefs, employment status, surgical history, or living arrangement. However, smoking status differed significantly (*χ*^2^ = 4.24, *p* = 0.039), with a higher proportion of smokers among patients who discontinued (34.8%) than among those who completed the program (14.3%). Additionally, the length of hospital stay prior to PAC enrollment was significantly shorter in the withdrawn group (mean = 22.70 days, SD = 9.19) than in the completed group (mean = 40.05 days, SD = 4.10; *Z* = −6.73, *p* < 0.001).

### 3.3. Comparisons of Muscle Strength, Physical Performance, and Functional Recovery Between and Within Groups

Table 2 presents the assessments of muscle strength, physical performance, functional recovery, nutritional status, cognitive function, occupational performance, and language ability at baseline, 3 weeks, and 7 weeks post-intervention. No significant between-group differences were observed at baseline for most functional measures—such as the mRS, BI, IADL, BBS, FOIS, MNA, MMSE, and occupational performance, except for affected upper limb muscle strength (*Z* = −2.00, *p* = 0.046), affected lower limb muscle strength (*Z* = −2.02, *p* = 0.043), gait speed (*Z* = −3.16, *p* = 0.002), the 6MWT (*Z* = −3.42, *p* < 0.001) and FMA-Sensory (*Z* = −2.27, *p* = 0.023), with the completed group showing poorer performance in these domains.

As shown in Table 2, significant improvements were observed across multiple domains within the completed group over time. Muscle strength demonstrated significant gains in both the affected and unaffected limbs by week 7. Physical performance also improved, with marked increases in the BBS scores, gait speed, and 6MWT scores by week 7. Functional recovery, as measured by BI and IADL, showed significant improvement at both 3 and 7 weeks. Additionally, substantial gains were observed in the Sensory and Upper Extremity domains of the FMA. Although detailed nutritional analysis was not the focus of this comparison, FOIS and MNA scores were included. Cognitive function and occupational performance significantly improved by week 7. In contrast, the withdrawal group exhibited no significant changes between baseline and 3 weeks across all outcome measures.

At the 3-week follow-up, although between-group comparisons revealed significant differences in gait speed (*Z* = −2.14, *p* = 0.032) and the 6MWT (*Z* = −2.28, *p* = 0.022)—favoring the withdrawn group, who demonstrated better initial ambulatory performance—no significant differences were found in the mean changes from baseline to week 3 across all outcome measures, indicating that the magnitude of short-term improvement was similar in both groups.

### 3.4. Effects of the PAC Rehabilitation Program Based on GEE Models

As shown in Table 3, the effects of the PAC program were assessed using GEE models to analyze data from patients who completed the program, with outcomes evaluated at three time points: baseline, week 3, and week 7. The models were adjusted for potential covariates, including age, sex, marital status, caregiver, religious belief, current employment, history of surgery, living status, stroke type, smoking, alcohol consumption, and comorbidities, and revealed significant improvements across multiple domains.

Muscle strength improved significantly at 7 weeks, with notable gains observed in the affected upper (B = 0.93, *p* < 0.001) and lower limbs (B = 0.88, *p* < 0.001), as well as in the unaffected upper (B = 0.32, *p* < 0.001) and lower limbs (B = 0.33, *p* = 0.006) (Table 3).

Physical performance also demonstrated significant improvements. The BBS score improved at both 3 weeks (B = 6.95, *p* = 0.016) and 7 weeks (B = 9.70, *p* = 0.003). Gait speed increased at 3 weeks (B = 0.10, *p* = 0.044) and 7 weeks (B = 0.23, *p* = 0.024). Additionally, the 6MWT improved at 3 weeks (B = 43.6, *p* = 0.011) and 7 weeks (B = 67.6, *p* = 0.001).

Functional recovery was improved significantly, as reflected by a decrease in the mRS at 7 weeks (B = −0.13, *p* = 0.028). The BI scores improved at both 3 weeks (B = 10.8, *p* < 0.001) and 7 weeks (B = 19.5, *p* < 0.001). IADL scores also improved significantly at 3 weeks (B = 1.02, *p* < 0.001) and 7 weeks (B = 1.48, *p* < 0.001). Significant improvements were also observed in the FMA, including both the sensory domain (3 weeks: B = 5.96, *p* = 0.018; 7 weeks: B = 10.2, *p* < 0.001) and the upper extremity motor domain (3 weeks: B = 8.06, *p* = 0.008; 7 weeks: B = 11.8, *p* < 0.001).

Regarding nutritional status, the FOIS score increased significantly at 7 weeks (B = 0.85, *p* = 0.012). Cognitive function also improved at 7 weeks, as indicated by the MMSE score (B = 3.36, *p* = 0.020). Finally, occupational performance quality improved significantly at both 3 weeks (B = 0.48, *p* = 0.026) and 7 weeks (B = 0.93, *p* < 0.001).

## 4. Discussion

This study explored the effectiveness of a 7-week PAC rehabilitation program for patients with stroke, focusing on improvements in muscle strength, physical performance, functional recovery, nutritional status, cognitive function, and occupational performance. Our findings demonstrated significant improvements across multiple domains among participants who completed the PAC program, highlighting its clinical efficacy and potential benefits for stroke recovery.

The completion rate of the PAC program in our study was 70.9%, which is higher than that reported in previous studies—for example, a mean adherence rate of 68.6% in China [35] and adherence rates of 49% pre-intervention and 54% post-intervention in Australia [36]. These findings suggest generally good adherence rates to post-acute rehabilitation programs. Notably, 23 participants (29.1%) in our cohort withdrew from the PAC program for various reasons, including death (*n* = 2), clinical deterioration requiring intensive care unit admission (*n* = 3), and sufficient functional improvement allowing for earlier discharge to home (*n* = 18). The overall withdrawal rate (29.1%) was significantly associated with shorter initial hospitalization and a higher prevalence of smoking. Previous research has revealed that individuals who smoke are less likely to access or complete rehabilitation programs after a cardiac event [37]. This association highlights the importance of targeted interventions focused on smoking cessation among stroke survivors to enhance rehabilitation adherence and outcomes.

Our findings demonstrated significant between-group differences in gait speed and 6MWT outcomes at both baseline and 3 weeks. Specifically, patients who discontinued the PAC program had faster gait speeds and greater 6MWT distances than those who completed the program. This suggests that non-compliance in some cases may have reflected early functional recovery and achievement of rehabilitation goals rather than poor adherence. These results are consistent with a large-scale longitudinal study based on the Third China National Stroke Registry, which found that faster gait speed at discharge and 3 months post-stroke was significantly associated with better long-term outcomes, including reduced risk of poor functional status, cognitive impairment, ischemic stroke recurrence, and composite vascular events at 1 year (OR range: 0.86–0.94; HR range: 0.92–0.94) [38]. This supports the notion that gait speed is not only a reliable functional measure but also a prognostic indicator of stroke recovery. From our clinical perspective, patients with higher initial functional capacity (as reflected by faster gait speed and longer walking distance) often demonstrate better balance, mobility, and self-care abilities early in the rehabilitation process. These individuals may reach discharge criteria ahead of schedule or prefer to transition to home-based recovery, leading to earlier discontinuation of the PAC program.

Improvements in muscle strength were particularly notable in both affected and unaffected limbs, suggesting that targeted rehabilitation can promote neuromuscular adaptations after stroke. These strength gains may play a critical role in enhancing physical function and reducing secondary complications, such as muscle atrophy and joint contractures. Recent studies have highlighted the effectiveness of resistance training in improving muscle strength and functional outcomes in patients with stroke. For instance, unilateral resistance training has been shown to significantly reduce muscle atrophy and enhance upper limb function, thereby promoting successful rehabilitation outcomes [39]. Additionally, focusing on the non-paretic side through strength training can enhance balance and mobility, potentially facilitating recovery on the affected side via neural plasticity mechanisms [40]. These findings underscore the importance of incorporating targeted resistance exercises into post-stroke rehabilitation programs to optimize patient outcomes.

Physical performance improvements were evident through significant gains in the BBS, gait speed, and the 6MWT. Enhancements in balance and gait are critical indicators of reduced fall risk and improved mobility among stroke survivors. Previous research has shown that persistent balance impairments following stroke are a major risk factor for increased fear of falling, a higher incidence of falls, and subsequently lower activity levels. Stroke survivors have been reported to experience falls at more than twice the rate observed in healthy individuals [41]. Our results further support a growing body of evidence indicating that structured rehabilitation programs can significantly improve balance and ambulatory function after stroke.

Functional recovery, assessed using the BI, IADL, and FMA for sensory and upper extremity function, significantly improved at both the 3-week and 7-week follow-ups. These improvements reflect the effectiveness of the structured PAC program in enhancing independence in daily life among patients with stroke, consistent with existing literature [4,42]. Notably, participants also demonstrated marked improvements in the mRS scores, indicating a reduction in overall disability. These findings underscore the importance of early and intensive rehabilitation interventions within the post-stroke continuum of care [43,44].

Additionally, improvements in nutritional status, swallowing ability, and cognitive function were observed at the 7-week follow-up. These findings align with prior studies emphasizing the importance of structured cognitive and nutritional rehabilitation in promoting recovery after stroke. Such programs typically include comprehensive cognitive assessments, individualized goal setting, targeted cognitive training, implementation of compensatory strategies, environmental modifications, structured repetition and practice, continuous monitoring with feedback, and, ultimately, the integration of cognitive strategies into daily life [45]. This underscores the importance of providing sufficient time and structured protocols to optimize cognitive recovery post-stroke.

In the present study, although the quality of movement in the affected upper limbs improved, the amount of use showed a limited gain, suggesting a potential gap between functional recovery and actual use in daily life. Recent studies have indicated that even when patients demonstrate good performance in upper limb function assessments, they may continue to underuse the affected limb due to a lack of confidence or motivation—a phenomenon known as “learned nonuse [46].” Furthermore, self-efficacy and movement quality have been identified as key predictors of upper limb usage frequency [47]. Therefore, future rehabilitation programs should incorporate strategies aimed at enhancing self-efficacy and providing individualized interventions to encourage active use of the affected limb in daily activities, thereby promoting more comprehensive functional recovery.

In the current study, language function did not significantly improve after the 7-week PAC rehabilitation program. This outcome may be attributed to the relatively short duration and moderate intensity of the speech and language therapy provided. According to a comprehensive Cochrane review, speech and language therapy can improve functional communication, reading, writing, and expressive language in individuals with post-stroke aphasia when delivered at sufficient intensity and duration [48]. However, high-dose interventions may lead to higher dropout rates and may not be suitable for all patients. These findings highlight the need for individualized speech and language therapy plans with tailored intensity and duration based on aphasia severity to optimize language recovery in future PAC protocols.

This study has some limitations. First, this retrospective study was conducted at a single hospital in Taiwan, which limits generalizability owing to potential selection bias arising from a non-representative sample and the influence of the inclusion and exclusion criteria. Additionally, the short 7-week follow-up period restricts the ability to assess the long-term effects of the PAC program. Second, the relatively small sample size, drawn from urban areas and a single hospital system, further limits the applicability of the findings to broader populations. Third, the use of a single-group pre-post design without a control group limits the ability to attribute observed improvements solely to the intervention. Factors, such as natural maturation, spontaneous recovery, regression to the mean, increased attention from healthcare providers, or other external influences, may have contributed to the results. While the inclusion of a control group could mitigate these concerns, it was deemed ethically inappropriate in this context, as withholding potentially beneficial interventions would raise ethical issues. However, future research may consider using matched cohort designs or historical controls to enhance the validity and generalizability of these findings. In addition, although cognitive function was assessed using the MMSE, the relationship between baseline cognition and functional prognosis was not specifically analyzed and warrants further investigation in future studies.

Despite the study’s limitations, our findings provide strong evidence supporting the effectiveness of structured PAC programs in improving functional recovery, physical performance, nutritional status, cognitive function, occupational performance, and muscle strength in patients with stroke. These findings highlight the substantial clinical benefits of comprehensive rehabilitation programs and reinforce their critical role in post-stroke recovery. Furthermore, this study identifies key areas for further investigation and highlights opportunities for optimizing clinical practice to maximize patient outcomes.

These overall improvements across multiple functional domains—ranging from muscle strength to physical performance, functional recovery, sensory and motor functions, swallowing, cognitive function, and occupational performance—can be attributed to the structured, intensive, and multidisciplinary nature of the PAC program. By initiating early, personalized interventions during the critical window of neuroplasticity, the program likely enhanced motor relearning and cognitive adaptation. The collaborative involvement of physicians, physical therapists, occupational therapists, speech–language pathologists, nutritionists, and nurses enabled comprehensive patient management tailored to individual needs. This team-based approach not only facilitated functional recovery but also addressed complex comorbidities and psychosocial barriers, reinforcing its value in optimizing holistic stroke rehabilitation. These mechanisms provide a plausible explanation for the broad functional gains observed in this study.

## 5. Conclusions

A structured PAC rehabilitation program demonstrated beneficial effects on muscle strength, physical performance, functional recovery, and occupational performance in patients at the post-acute stage of stroke. Leveraging this critical window—often referred to as the “golden period” of rehabilitation—can significantly impact recovery trajectories. Future studies employing more rigorous designs and longer follow-up periods are recommended to evaluate long-term functional outcomes, including social reintegration and return to work.

## Figures and Tables

**Table 1:** Comparisons of baseline sociodemographic and disease characteristics between patients who completed and those who discontinued the PAC program.

Variable	All (*n* = 79)	Completed (*n* = 56)	Discontinued (*n* = 23)	*x*^2^/*Z*	*p* *
	Mean (SD)/*n* (%)	Mean (SD)/*n* (%)	Mean (SD)/*n* (%)		
*Stroke type*				1.81 ^a^	0.215
Hemorrhagic stroke	14 (17.7)	12 (21.4)	2 (8.7)		
Ischemic stroke	65 (82.3)	44 (78.6)	21 (91.3)		
*Sociodemographic*					
Age (years)	67.35 (13.62)	66.00 (13.25)	70.65 (14.24)	−1.46	0.143
Sex				0.01 ^a^	0.910
Men	42 (53.2)	30 (53.6)	12 (52.2)		
Women	37 (46.8)	26 (46.4)	11 (47.8)		
Caregiver				0.01 ^a^	0.994
Family members	38 (48.1)	27 (48.2)	11 (47.8)		
Foreign domestic worker	21 (26.6)	15 (26.8)	6 (26.1)		
Professional caregiver	20 (25.3)	14 (25.0)	6 (26.1)		
Marital status				3.13 ^a^	0.373
Single/divorced/widowed	16 (20.3)	12 (21.4)	4 (17.4)		
Married	63 (79.7)	44 (78.6)	19 (82.6)		
Religious belief	41 (51.9)	31 (55.4)	10 (43.5)	2.13 ^a^	0.831
Currently employed	26 (32.9)	20 (35.7)	6 (26.1)	2.30 ^a^	0.890
Operation history	59 (74.7)	41 (73.2)	18 (78.3)	5.47 ^a^	0.362
*Living status*				0.07 ^a^	0.787
Alone	8 (10.1)	6 (10.7)	2 (8.7)		
Living with families	71 (89.9)	50 (89.3)	21 (91.3)		
*Lifestyle*					
Current smoker	16 (20.3)	8 (14.3)	8 (34.8)	4.24 ^a^	0.039
Alcohol consumption	12 (15.2)	8 (14.3)	4 (17.4)	0.12 ^a^	0.727
*Length of stay* (days)	35.00 (9.93)	40.05 (4.10)	22.70 (9.19)	−6.73	<0.001
*Comorbidities*					
Hypertension	55 (69.6)	40 (71.4)	15 (65.2)	0.30 ^a^	0.586
Heart disease	21 (26.6)	15 (26.8)	6 (26.1)	0.01 ^a^	0.949
Type 2 diabetes	31 (39.2)	21 (37.5)	10 (43.5)	0.24 ^a^	0.621
Lipidemia	5 (6.3)	3 (5.4)	2 (8.7)	0.31 ^a^	0.580
*Muscle strength*					
Affected upper limb	2.71 (1.40)	2.52 (1.41)	3.17 (1.27)	−2.00	0.046
Affected lower limb	3.03 (1.30)	2.86 (1.33)	3.43 (1.16)	−2.02	0.043
Unaffected upper limb	4.53 (0.71)	4.52 (0.76)	4.57 (0.59)	−0.14	0.888
Unaffected lower limb	4.34 (0.83)	4.38 (0.87)	4.26 (0.75)	−0.90	0.368
*Physical performance*					
BBS (range: 0–56)	23.97 (21.08)	23.60 (21.08)	24.83 (21.53)	−0.51	0.608
Gait speed (m/s)	0.26 (0.33)	0.21 (0.33)	0.47 (0.26)	−3.16	0.002
6MWT (meters)	87.17 (110.56)	68.85 (101.78)	172.67 (113.90)	−3.42	<0.001
*Functional recovery*					
mRS (range: 0–6)	3.87 (0.34)	3.88 (0.33)	3.87 (0.34)	−0.07	0.948
3	10 (12.7)	7 (12.5)	3 (13.0)	0.01 ^a^	0.947
4	69 (87.3)	49 (87.5)	20 (87.0)		
Barthel Index (range: 0–100)	38.18 (24.01)	35.80 (21.68)	45.56 (29.65)	−0.99	0.320
IADL	1.36 (1.44)	1.37 (1.42)	1.32 (1.52)	−0.22	0.826
Male (range: 0–5)	1.61 (1.61)	1.63 (1.56)	1.55 (1.81)	−0.29	0.773
Female (range: 0–8)	1.06 (1.16)	1.04 (1.16)	1.09 (1.22)	−0.13	0.896
FMA-Sensory (Range: 0–44)	23.55 (17.64)	21.14 (17.81)	29.95 (15.81)	−2.27	0.023
FMA-Upper extremity (Range: 0–66)	29.00 (21.86)	26.78 (21.50)	34.30 (22.29)	−1.54	0.125
*Nutritional status*					
FOIS (range: 1–7)	4.92 (2.41)	5.02 (2.45)	4.70 (2.36)	−0.42	0.675
MNA (range: 0–30)	11.78 (1.72)	11.64 (1.86)	12.13 (1.25)	−0.99	0.324
*Cognitive function*					
MMSE (range: 0–30)	20.05 (10.29)	19.41 (10.93)	21.85 (8.24)	−0.84	0.400
*Occupational performance*					
Amount of use (range: 0–5)	2.87 (6.25)	3.33 (7.28)	1.70 (1.56)	−0.74	0.462
Quality of movement (range: 0–5)	1.20 (1.56)	1.03 (1.53)	1.64 (1.58)	−1.70	0.089
*Language ability*					
CCAT (range: 0–12)	8.25 (3.75)	8.42 (3.85)	7.65 (3.42)	−1.36	0.174

Note: * *p*-values represent comparisons between patients who completed and those who discontinued the PAC program. ^a^ Fisher’s exact test was applied. BBS: Berg balance scale; 6MWT: 6-minute walk test; mRS: modified Rankin scale; IADL: Instrumental Activities of Daily Living; FMA-Sensory: Fugl–Meyer sensory assessment; FMA-Upper extremity: Fugl–Meyer upper assessment; FOIS: functional oral intake scale; MNA: mini nutritional assessment; MMSE: mini-mental state examination; CCAT: concise Chinese aphasia test.

**Table 2:** Comparison of muscle strength, physical performance, and functional recovery within and between groups.

	Completed (*n* = 56)							Discontinued (*n* = 23)				Between-Group					
Parameter	Baseline	3-Week	7-Week	Baseline vs. 3-Week		Baseline vs. 7-Week		Baseline	3-Week	Baseline vs. 3-Week		Baseline		3-Week		3-Week vs. Baseline Mean Differences	
				*t*	*p*	*t*	*p*			*t*	*p*	*Z*	*p* *	*Z*	*p* *	*Z*	*p* *
*Muscle strength*																	
Affected side																	
Upper limb	2.52 (1.41)	3.00 (1.27)	3.56 (1.42)	−1.90	0.060	3.79	<0.001	3.17 (1.27)	3.15 (1.21)	0.05	0.963	−2.00	0.046	−0.29	0.771	−0.21	0.831
Lower limb	2.86 (1.33)	3.21 (1.20)	3.82 (1.13)	−1.49	0.138	3.99	<0.001	3.43 (1.16)	3.23 (1.17)	0.51	0.616	−2.02	0.043	−0.14	0.888	−1.04	0.300
Unaffected side																	
Upper limb	4.52 (0.76)	4.77 (0.57)	4.86 (0.41)	−1.96	0.052	2.93	0.004	4.57 (0.59)	4.54 (0.66)	0.13	0.901	−0.14	0.888	−1.59	0.111	−0.90	0.369
Lower limb	4.38 (0.87)	4.54 (0.79)	4.70 (0.71)	−1.03	0.305	2.13	0.036	4.26 (0.75)	4.08 (1.12)	0.59	0.558	−0.90	0.368	−1.79	0.073	−0.04	0.967
*Physical* *performance*																	
BBS (score)	23.60 (21.08)	31.37 (21.55)	33.79 (21.85)	−1.88	0.063	2.38	0.020	24.83 (21.52)	30.42 (24.35)	−0.70	0.490	−0.51	0.608	−0.22	0.829	−0.72	0.470
Gait speed (m/s)	0.21 (0.33)	0.31 (0.36)	0.48 (0.78)	−1.48	0.143	2.15	0.035	0.47 (0.26)	0.62 (0.38)	−1.10	0.284	−3.16	0.002	−2.14	0.032	−1.75	0.080
6MWT (meters)	68.85 (101.78)	111.46 (131.16)	148.88 (156.08)	−1.92	0.058	3.02	0.003	172.67 (113.90)	239.25 (154.75)	−1.11	0.281	−3.42	<0.001	−2.28	0.022	−1.73	0.084
*Functional* *recovery*																	
mRS	3.88 (0.33)	3.82 (0.43)	3.75 (0.51)	0.74	0.464	−1.53	0.130	3.87 (0.34)	3.74 (0.54)	0.98	0.335	−0.07	0.948	−0.63	0.531	−1.16	0.244
Barthel Index	35.80 (21.68)	49.04 (26.76)	57.10 (28.98)	−2.88	0.005	4.24	<0.001	45.56 (29.65)	50.56 (33.68)	−0.40	0.696	−0.99	0.320	−0.01	0.992	−0.47	0.638
IADL	1.37 (1.42)	2.49 (1.96)	2.98 (2.18)	−3.33	0.001	4.32	<0.001	1.32 (1.52)	2.36 (2.06)	−1.65	0.109	−0.22	0.826	−0.25	0.799	−0.53	0.596
FMA-Sensory	21.14 (17.81)	28.88 (17.87)	33.65 (15.28)	−2.29	0.024	3.87	<0.001	29.95 (15.81)	31.18 (17.08)	−0.20	0.840	−2.27	0.023	−0.27	0.786	−0.99	0.324
FMA-Upper extremity	26.78 (21.50)	36.55 (22.06)	39.98 (22.32)	−2.36	0.020	3.09	0.003	34.30 (22.29)	41.83 (25.00)	−0.91	0.369	−1.54	0.125	−1.08	0.281	−1.17	0.241
*Nutritional status*																	
FOIS	5.02 (2.45)	5.68 (2.42)	5.80 (1.90)	−1.44	0.154	1.85	0.067	4.70 (2.36)	5.33 (2.15)	−0.78	0.440	−0.42	0.675	−0.42	0.676	−0.04	0.971
MNA	11.64 (1.86)	11.70 (2.24)	12.08 (1.66)	−0.14	0.891	1.27	0.208	12.13 (1.25)	12.25 (1.14)	−0.28	0.784	−0.99	0.324	−0.47	0.638	−0.28	0.778
*Cognitive function*																	
MMSE	19.41 (10.93)	22.25 (9.97)	24.00 (6.56)	−1.44	0.154	2.63	0.010	21.85 (8.24)	22.55 (7.45)	−0.23	0.818	−0.84	0.400	−0.09	0.932	−0.25	0.806
*Occupational* *performance*																	
Amount of use	3.33 (7.28)	3.08 (6.03)	2.03 (2.03)	0.20	0.844	−1.28	0.204	1.70 (1.56)	2.08 (2.10)	−0.56	0.584	−0.74	0.462	−0.06	0.950	−0.42	0.678
Quality of movement	1.03 (1.53)	1.63 (1.82)	2.07 (2.03)	−1.91	0.059	2.95	0.004	1.64 (1.58)	2.08 (2.10)	−0.66	0.520	−1.70	0.089	−0.42	0.673	−0.52	0.605
*Language ability*																	
CCAT	8.42 (3.85)	8.96 (3.47)	9.34 (3.35)	−0.78	0.438	1.30	0.196	7.65 (3.42)	7.64 (3.60)	0.00	0.999	−1.36	0.174	−1.42	0.157	−0.489	0.625

Note: Data are presented as mean (SD); * *p* value derived from Mann–Whitney U test for between-group comparisons. BBS: Berg balance scale; 6MWT: 6-minute walk test; mRS: modified Rankin scale; IADL: Instrumental Activities of Daily Living; FMA-Sensory: Fugl–Meyer sensory assessment; FMA-Upper extremity: Fugl–Meyer upper assessment; FOIS: functional oral intake scale; MNA: mini nutritional assessment; MMSE: mini-mental state examination; CCAT: concise Chinese aphasia test.

**Table 3:** Effectiveness of the post-acute-care rehabilitation program on muscle strength, physical performance, and functional recovery in stroke patients based on GEE models (*n* = 56).

Parameter		B	SE	95% CI		*p*
				Lower	Upper	
*Muscle strength*						
Affected side						
Upper limb						
	3-week	0.31	0.20	−0.08	0.70	0.115
	7-week	0.93	0.22	0.49	1.36	<0.001
Lower limb						
	3-week	0.19	0.18	−0.16	0.54	0.28
	7-week	0.88	0.18	0.52	1.24	<0.001
Unaffected side						
Upper limb						
	3-week	0.18	0.10	−0.01	0.37	0.069
	7-week	0.32	0.09	0.15	0.50	<0.001
Lower limb						
	3-week	0.09	0.12	−0.16	0.33	0.489
	7-week	0.33	0.12	0.09	0.57	0.006
*Physical performance*						
BBS						
	3-week	6.95	2.88	1.32	12.6	0.016
	7-week	9.70	3.26	3.32	16.1	0.003
Gait speed (m/s)						
	3-week	0.10	0.05	0.00	0.20	0.044
	7-week	0.23	0.10	0.03	0.44	0.024
6MWT (meters)						
	3-week	43.6	17.20	9.88	77.3	0.011
	7-week	67.6	20.5	27.4	108	0.001
*Functional recovery*						
mRS						
	3-week	−0.08	0.05	−0.18	0.03	0.159
	7-week	−0.13	0.06	−0.24	−0.01	0.028
Barthel Index						
	3-week	10.8	3.14	4.62	16.9	0.001
	7-week	19.5	3.66	12.3	26.7	<0.001
IADL						
	3-week	1.02	0.23	0.56	1.48	<0.001
	7-week	1.48	0.28	0.93	2.03	<0.001
FMA-Sensory						
	3-week	5.96	2.52	1.03	10.9	0.018
	7-week	10.2	2.65	5.03	15.4	<0.001
FMA-Upper extremity						
	3-week	8.06	3.06	2.07	14.0	0.008
	7-week	11.8	3.37	5.21	18.4	<0.001
*Nutritional status*						
FOIS						
	3-week	0.65	0.35	−0.03	1.34	0.061
	7-week	0.85	0.34	0.19	1.51	0.012
MNA						
	3-week	−0.02	0.27	−0.55	0.51	0.934
	7-week	0.24	0.25	−0.26	0.73	0.355
*Cognitive function*						
MMSE						
	3-week	2.13	1.47	−0.75	5.00	0.147
	7-week	3.36	1.45	0.52	6.19	0.02
*Occupational performance*						
Amount of use (AOU)						
	3-week	−0.07	0.89	−1.81	1.66	0.933
	7-week	−0.96	0.77	−2.48	0.56	0.216
Quality of movement (QOM)						
	3-week	0.48	0.21	0.06	0.90	0.026
	7-week	0.93	0.25	0.44	1.42	<0.001
*Language ability*						
CCAT						
	3-week	0.50	0.57	−0.61	1.61	0.380
	7-week	0.98	0.60	−0.20	2.15	0.105

Note: B: unstandardized regression coefficient; SE, standard error; CI: confidence interval; BBS: Berg balance scale; 6MWT: 6-minute walk test; mRS: modified Rankin scale; IADL: Instrumental Activities of Daily Living; FMA-Sensory: Fugl–Meyer sensory assessment; FMA-Upper extremity: Fugl–Meyer upper assessment; FOIS: functional oral intake scale; MNA: mini nutritional assessment; MMSE: mini-mental state examination; CCAT: concise Chinese aphasia test; models were adjusted for age, sex, marriage, caregiver, religious beliefs, current employment, ever having an operation, living status, stroke type, smoking, alcohol consumption, comorbidities (hypertension, heart disease, type 2 diabetes, lipidemia, etc.). Reference group: baseline.

## Data Availability

The data used and analyzed during the current study are available from the corresponding author upon reasonable request.

## Abbreviations

BBS: Berg Balance Scale; BI: Barthel Index; FMA: Fugl–Meyer Assessment; IADL: Instrumental Activities of Daily Living; ICU: intensive care unit; mRS: modified Rankin Scale; PAC: post-acute care; 6MWT: 6-Minute Walk Test.
